# Movement as a Somaesthetic Source of Subjectivity

**DOI:** 10.3389/fpsyg.2021.688296

**Published:** 2021-08-30

**Authors:** Robert Dobrowolski, Krzysztof Pezdek

**Affiliations:** Department of Physical Education and Sport Sciences, University School of Physical Education in Wrocław, Wrocław, Poland

**Keywords:** body, movement, phenomenology, psychoanalysis, aesthetics

## Abstract

The paper discusses two opposite understandings of how the kinaesthetic experience of movement translates into the development of subjectivity. One of them, in which somatically experienced movement is regarded as a positive source of authentic self-fashioning, will be described within the framework of phenomenology. The other, which emphasises the inauthentic nature of movement, will be described in term of psychoanalysis. Subsequently, the two opposite interpretations will be discussed in the conciliatory perspective of aesthetic experience, in which the contradiction of spontaneity and conformity will be shown as a quasi-artistic factor which bolsters the dynamics of subjectivity development.

## Introduction

The philosophical imperative of seeking fully rational knowledge is usually associated with an utter repudiation of the body; allegedly, the thinking subject must first shake off the yoke of the senses and go out of the Platonic cave of appearances into the full light of abstract reason in order to eventually perceive the unchangeable truth of him/herself and the world in the environment of pure concepts, which is uncontaminated by mutability. If “in the beginning was the Word,” there is no point looking for a different path to the source of knowledge than the one which the logos of language charts amidst the murky realm of nature.

In ancient Greece, contempt for the sensory *doxa* – for the erroneous belief engendered in the chaos of empiricism – reached its apex in the teachings of Zeno of Elea. In their aversion to corporally engendered knowledge, Zeno and his followers, referred to as the Eleatics, did not hesitate to negate movement as a sensory illusion which contravened the rational unchangeability of truth. An oft-repeated anecdote of old has it that instead of engaging in a protracted discussion with Zeno, one of his opponents began to pointedly walk to and fro in front of him. While he was totally silent, he embodied the living truth in an eloquent and uncontestably visible manner.

This dispute has been rehearsed till the present day. The difference is that today it unfolds as rivalry between “anachronistic” experiences of the analogue body and knowledge produced by cybernetic simulations. We can see that on the daily basis, for example when we find it increasing difficult to meander our way through a growing crowd of spectral strollers who are entirely immersed in their smartphones. This forces the realisation that in the confrontation is commandingly won by the digitally disembodied utopia, which promises an ultimate liberation from this mortal coil in virtual paradises which proliferate at a staggering pace. We hold a grudge against these strollers, not only because they can no longer move like humans among other humans, but also because their virtual separation fosters a narcissistic incapacity to think properly and to experience things in the first place. Their flawed manner of moving appears to be a symptom and simultaneously a cause of their mental deformity.

Apparently, the deficit of right movement is not only a reason behind the commonly diagnosed and discussed obesity epidemic, but also a cause of uniquely mental underweight. Namely, a cybernetically mediatised individual is emptied of the weight of psychosomatic being, as his/her virtual “lightness of being” dissolves his/her capacity to respond to and act in conformity with the tough rules of tangible reality, which results in the subject becoming a foreign body in the universe of Nature. Increasingly disoriented and confused in the virtual, contemporary people probably owe it to their natural bodiliness that they have not lost ground beneath their feet yet. Admittedly, Foucault insisted that “[n]othing in man – not even his body – is sufficiently stable to serve as the basis for self-recognition or for understanding other men” (Foucault, 1977, p. 153), but mindful receptivity to body awareness does not have to degenerate into a perennially suspect cult of natural bodiliness. Does the tactile–kinaesthetic body itself not encourage us to adopt a more flexible attitude, warning us against fundamentalism as a leaning that in the long run produces the hazard of crippling rigidity?

This paper discusses two opposite understandings of how the kinaesthetic experience of movement translates into the development of subjectivity. One of them, in which somatically experienced movement is treated as a positive source of authentic self-fashioning, will be discussed within the framework of phenomenology and phenomenologically inspired neurology. The other, which emphasises the inauthentic and unconsciously imitative nature of both experienced and represented movement, will be described in terms of Jacques Lacan’s psychoanalytical model, primarily referring to his concept of the “mirror stage,” whose relevance extends beyond psychoanalysis and which has been appreciatingly adopted in multiple theories of individual development. The two opposing interpretations will then be discussed from the conciliatory perspective of aesthetic experience, where the contradiction between spontaneity and conformity invigorates the dynamics of quasi-artistic subjectivity development, adding up to a somaesthetic game. As this game is not played for any mythical essence of personality, what is really at stake in it is a harmoniously and completely embodied existence.

## Movement as a Natural Source of Subjectivity

If we explore the world from an ego-centric perspective and equate its emergence with our own birth, we can certainly conclude that movement lay at its very beginning. It is from this primal movement that everything that exists has arisen. Similarly, the soulful *homo sapiens* first experiences him/herself and the world as a body-in-motion. Goethe’s Faust was broadly off the mark as his assertion that “in the beginning was the deed” failed to capture the actual chronology of things. It is only the sensation of movement that brings forth the subject of the “deed,” and it is from this subject’s bodily activity that the subject of the “word” is subsequently formed. This was exactly what Edmund Husserl concluded from his phenomenological analyses: “Originally the ‘I move’ […] precedes the ‘I can do”’ (Husserl, 2000, p. 273).

However, before we consciously begin to move ourselves and objects of the external world, our primal sensibility is first engrossed by movement as such, in its sheer manifestation, by the yet un-subjected motion-in-self, an element from which a future subject will soon arise as if from a dynamic matrix. This yet un-subjected movement is by no means perceived as objective movement. It is by no means about any objectively observable change in space, but about bodily movement experienced by the lived body, such as when in the kinaesthetic process of self-cognisance, an infant of and by itself begins to move its eyes, even though its sight does not follow any particular object.

Clearly, our first consciousness is a tactile-kinesthetic consciousness that arises on the ground of movement that comes to us spontaneously, indeed, on the ground of fundamental and invariant species-specific kinetic acts that we simply “do” in coming into the world, acts such as kicking, stretching, sucking, swallowing, and so on. Such acts happen to us before *we* make them happen. In just this sense, movement is there prior to “I move.” Kicking, for example, is there before *I* kick; stretching is there before *I* stretch. In effect, *movement forms the I that moves before the I that moves forms movement*. (Sheets-Johnstone, 2011, pp. 118–119)

Studies on infants have found that infants aged between 6 and 22 weeks old are “not affected by feature differences. For them movement is predominant. They respond to a change in motion but not to a change in size, shape, or colour” (Bower, 1971, p. 37). Bower adds that it means that very young babies “ignore features to such an extent that I would suggest they respond *not to moving objects but to movements*” (Bower, 1971, p. 37).

Developmental psychology research indicates that we already sense ourselves as really acting agents in early infancy. We turn to what is outside, we twist toward objects, reach for them, push them away, touch them, etc. We move inside with equal energy as well, when crying, swallowing, sucking, chewing, etc.

As observed by infant psychiatrist Daniel N. Stern, over the first 2 months of infants’ lives, when they are in “some kind of presocial, precognitive, preorganised life phase” (Stern, 1985, p. 37), processes unfold in which they acquire a sensory self-awareness.

Highlighting the meaning-producing role of the body and its primary semantics, from which the metaphors and metonymies of later language ensue, some theorists of the sensorimotor origin of subjectivity even insist that there is a “muscle sense.” They recognise muscle contractions and relaxations as the most elementary aspects of self-knowledge and the knowledge of the external world.

In his history of the muscle sense and of innervation feelings, historian of science Eckart Scheerer points out the central (and largely overlooked) role of nineteenth-century philosopher Johann Jakob Engel who, as Scheerer notes, may be properly regarded “the father of the muscle sense.” […] Scheerer emphasizes Engel’s critical point that “feelings of innervation” – will, volition, and the like – are not *ideas*, as Locke and Hume affirm, but incipient bodily tensions. He furthermore calls attention to Engel’s insight – that “muscles are organs by which we acquire ideas about external objects.” (Sheets-Johnstone, 2011, p. 399)

The kinaesthetic apperception of movement is also a prerequisite for acquiring linguistic competences. A child must first recognise him/herself as an active source of sound and as a physical agent; otherwise, s/he will not be able to develop the skill of speaking or even the very wish to use speech. Early on, s/he effectively experiences him/herself as a corporally competent manipulator to later develop a related desire to capture the world even more powerfully, i.e., by means of aptly and accurately selected words. The primary awareness of one’s own power is not a linguistic achievement; on the contrary, it is a direct bodily sensation which is the source from which even the most basically conceived subject derives his/her elementary meaning.

How an increasing deficit of proprioception and kinaesthesia leads to an exacerbating feeling that one’s own being is becoming unreal was suggestively described as early as in the 1970s. Olivier Sacks’s “neurological bestseller” described a “disembodied lady” to illustrate such an existential degradation (Sacks, 1998, pp. 43–54). Sacks augmented the neurological diagnosis with Wittgenstein’s insights about the corporeal basis of certainty:

“If you do know that *here is one hand*, we’ll grant you all the rest.” But then, in the same breath, on the same opening page: “What we can ask is whether it can make sense to doubt it …”; and, a little later, “Can I doubt it? Grounds for *doubt* are lacking!” (Sacks, 1998, p. 43)

Body awareness not only precedes the later development of language skills, but also makes it possible. It enables us to somatically feel how we co-ordinate the perceptual input all by ourselves into a volitionally directed output, a response whose extralinguistic meaning is conveyed by motor actions. In this way, the tactile–kinaesthetic body is realised, while mental agency is recognised, as the co-ordination and direction of our actions in the world afford it opportunities for asserting itself in this world.

We discover that we exist not through thinking, or through acts of disembodied self-reflection, but through self-motion, which helps us differentiate ourselves from environmental motion. Given the fundamental relevance of this primal experience, we should rephrase the Cartesian “cogito ergo sum” into “I move therefore I am.”

Furthermore, it is through experiencing our own mobility that we develop a propensity for perceiving external moving objects as living entities. I and my world – a couple grappling with each other – begin their life affair without words.

When first words eventually appear, they will not come out of the blue; their meaning will first be forged in the corporeally experienced metaphorics of movement itself. Without the natural semantics of the body in motion without its sensual symbolism, the abstract speech of the intellect could not possibly take shape.

Our conceptual system is grounded in, neurally makes use of, and is crucially shaped by our perceptual and motor systems; moreover, major forms of rational inference are instances of sensorimotor inference. (Lakoff and Johnson, 1999, p. 555)

Even the most formal and sublime language resonates with tactile–kinaesthetic experiences. A considerable amount of movement and sensory phrasings can be found, for example, in the lofty pronouncements of mathematics and logic. A theorem is *equivalent*^1^; a given proof sequence *leads* to the *following* conclusions; a mode of reasoning *surmounts* another one; and the *edge* that yet another one has over them is that it takes fewer *steps* to *clarify* and *disentangle* a problem. The body-language relations are so far-ranging that even:

[t]he rhythmicity of one’s movements, the alternation of muscular tension and relaxation in movement, go together with the capacity for linguistic modulation and general musicality. (Reich, 1945–1972, p. 345)

If a foreign language is not kinaesthetically entrenched in a listener’s linguistic sensorimotor system, it will not be a language to him/her at all, merely coming across as a series of meaningless sounds. It can concern even apparently well-known texts, as was the case, for example, in the 1960s, when African singer Miriam Makeba’s “The Click Song” was a frequently played international hit. Makeba performed a traditional song of the Xhosa people of South Africa, in which some words or sounds, such as “x” and “q,” were replaced by specific deep-throat “clicks,” consistently with the Xhosa linguistic rules. Most of those who listened to this song back then are probably still unaware of their symbolic linguistic meanings. This is how foreign the kinaesthetics of the “click-words” is to them.

The kinaesthetic genesis of language and thinking is borne out even by such an intellectually inflected word as “concept,” which has retained its tactile–kinetic legacy in many languages (e.g., German *Begriff*, which is etymologically associated with *greifen* – to reach out, to grab; or Polish *pojêcie*, which is derived from *pojmaæ*, *schwytaæ* – to capture, to catch)^2^. This implies that even the most abstract articulations harbour distinctive traces of bodily influences. In order to grasp the world in thought and word, one must first capture it directly, physically and palpably. Hence, even when we think and talk about high-brow themes, we *follow* a certain line of reasoning, *crack* problems and *chew* thoughts over and over again until we *reach* the gist of the matter and *nail* the essence of it, by *striking* at the core and *weighing* each word not to let the best solution *slip* us.

That movement fascinates is nothing random. If living itself is the most important part of life, it should not come as a surprise that we are captivated by what is its most real and most genuine manifestation, that is, by movement. Inanimate things are like a dead stone – incapable of self-motion, completely inert, and non-sentient. As the developmental psychology of young children confirms, this is how our inborn, intuitive identification of life and movement works. This is also the reason why, as adults, we tend to thoughtlessly stare at a flickering TV screen for hours on end. Nailed to the “motion pictures,” entranced viewers fall victim, so to speak, to paradoxical escapism; specifically, they flee from so-called life in order to contemplate its primordial essence – incessantly pulsating movement.

Besides the perceptual privileging of objects in motion, the special link between perception and movement is rendered in the determination of our sensorics by the motor system of our bodies.

This is how this connection is expressed in contemporary enactivist theory:

[…] the cognitive activity depends on the possibilities of action of the body because the mind is inherent in the active body. Sensory and motor processes, perception and action, are inseparable in cognition. […] Cognitive structures emerge from the recurrent sensorimotor patterns that enable action to be perceptually guided. The mind, one’s knowledge about the world and linguistic meaning emerge as part of this activity. (Scarinzi, 2015, p. 266)

In his Noë (2004, pp. 7–11), eminent enactivist Alva Noë describes an experiment which powerfully corroborates the central tenet of enactivism, i.e., a strong interdependence between perception and the sensorimotor system. Participants in the experiment were given purpose-designed left–right reversing goggles, which inverted their field of vision. While initially their perception was disturbed due to incongruities between the sensory knowledge generated by respective individuals’ experiences and the visual effects of inversion, eventually the image became stabilised and, which surprised many, ultimately conformed to non-visual modalities of sensory experience, for example, to the proprioceptive input. The sensorimotor exploration of the setting contributed to eliminating the illusory effects of the optic inversion: despite the “reversed” retinal stimulation, both sides – left and right – returned to their proper, objective positions. The body-in-motion restored reality to its proper place.

## Movement as a Medium of Alienation

However, it is not through its direct link to the visual perception that movement is manifest in us as the source and a symptom of all subjective aliveness. Movement which is visually perceived as an external change of location is merely a mimetic opportunity for its tactile–kinetic reproduction. This primarily involves the capacities to identify with movement discerned outside and then to fully absorb the experience of it. These capacities are described in the theory of mirror neurons, which holds that watching somebody else’s movement is enough to activate the observer’s neurons that are involved in generating this movement.

Long before the concept of mirror neurons was developed, philosopher Merleau-Ponty (1964) had inquired, for example, how it was possible for infants, who had neither self-awareness nor capability of recognising their faces, to anyway reciprocate a smile. How do children so young know which facial muscles to move in order to produce such a mimetic effect. Tellingly, as established by Meltzoff and Moore (1983, pp. 702–709), infants are able to imitate adults’ tongue and facial motions a mere 42 min after birth.

Described by phenomenology and confirmed by neurological research, the identification with a moving body is also explored in an entirely different theoretical framework, namely, in psychoanalysis (De Preester and Knockaert, 2005; Weigel and Scharbert, 2016). In his elaboration on the psychoanalytical approach, Jacques Lacan developed the concept of the “mirror stage” (Lacan, 2006, pp. 75–81), i.e., a phase in which children between 6 and 18 months of age become fascinated with their mirror reflections and adopt these images as an ideal of their psychosomatic selves. This is a turning point in any individual’s subjective development. It can be briefly characterised in terms of the distinction between “body schema” and “body image,” notions from outside the typical psychoanalytical lexicon. While the distinction was admittedly made by Austrian psychoanalyst and neurologist Paul Ferdinand Schilder, today it is far more frequently applied in phenomenology and phenomenologically inspired neurological research. Among the multiple differing definitions coined in the field, the most useful and most distinctive one says that the body schema is understood as a sensitive awareness of one’s own body as a set of possibilities. For example, although we have not measured the distance between our right hand resting on the desktop and a waste paper basket standing next to the desk, we feel that without moving and bending over, we cannot touch this basket. For its part, the body image denotes both the current visual perception of one’s own body and one’s knowledge of it. For example, even if we are completely anaesthetised and blindfolded, our “body image” will anyway contain our knowledge that, for instance, we have two arms.

Relying on this terminology, we can say that in the mirror stage, a child’s body schema is confronted with his/her body image for the first time, and that his/her further development depends on the outcomes of this confrontation. In this confrontation, the body schema corresponds to the Lacanian Real, while the body image to the Lacanian Imaginary.

Lacan added the Symbolic to the Real and the Imaginary, insisting that these three orders together constituted the whole of reality that we experienced. In broad lines, the Symbolic, which is represented by the so-called Big Other^3^, is the linguistically formed sphere of the Law that is valid in a given culture. The Imaginary is founded on the identificatory relations of the ego and its “specular” equivalents. Characterised by a narcissistic mixture of fascination and aggression, these relations produce an illusion of mutual resemblance and reciprocity, as well as rivalry and the desire to fully master the imaginary other (Lacan, 1988). At the level of perception, the Imaginary works like Photoshop: it disguises all unsettling imperfections and makes perception familiar and tailored for us. The imaginary correction only fails when it encounters too strong a resistance from the Real. The point is that the Real cannot find its own place in the symbolically structured Imaginary and explodes its coherence as a foreign body, triggering anxiety and an acute sense of insecurity. The Real defies any complete symbolic integration into the imaginary field of perception.

In the mirror stage, a child feels his/her body in the dimension of the Real as an animation evading his/her control, which fragments his/her tactile–kinaesthetic body into chaotic sensations and uncoordinated motorics. Experienced in this way, the body schema aggravates the child’s frustration caused by the separation from the “maternal” body^4^. This is the reason why the specular body image makes such a powerful impression on the child: a clear-cut figure emphasising his/her independence from the environment, and especially the view of this figure in motion which the child seems to have in control as a completely integrated unity. As a result, the palpably felt “body-in-pieces” of a child who is fascinated with his/her mirror reflection is ousted by the ideal of bodily autonomy. The other perceived in the mirror becomes an imaginary prop for turning the child’s own body into a “live” symbol of separateness, and thus the body becomes his/her first “own” signifier for his/her future, linguistified “Self.” This is how the somatic begins to align with the symbolic. In this way, the subject’s real body undergoes spectacular idealisation, for as “Lacan argues […] it is through the Imaginary register that we idealise particular bodies over others, and because the Imaginary is the register of spectacle and display, the visual field is thus where idealisations are formed” (Alpha and Hurst, 2018, p. 187).

Besides providing a sensory integration model which kindles hope for filling the gap left by the maternal body and for regaining control of the self and the surrounding world, the specular other also triggers aggression resulting from the inability of the still fragmented and non-autonomous narcissus to fully assimilate it. Both specular-imaginary stimuli – fascination and fear – contribute to the process in which the prior “body-in-pieces” schema is suffused with and taken over by the mirror image of the ideal body:

The imaginary exerts a captivating power over the subject, founded in the almost hypnotic effect of the specular image. The imaginary is thus rooted in the subject’s relationship to his own body (or rather to the image of his body). This captivating/capturing power is both seductive […] and disabling: it imprisons the subject in a series of static fixations […]. (Evans, 2006, p. 84)

In the typical course of psychosomatic development, the imaginarily and symbolically specified body eventually transforms into the phallic signifier of self-mastery and mastery of the world. As its realness is appropriated by the imaginary-symbolic coloniser, the body becomes an object of total surveillance and training and begins to move like a puppet manipulated by the Big Other. Thus it is not the spontaneously acting subject, but rather the subject-impersonating spirit of the symbolic law that is the primary mover of such a body:

In other words, the idea of a presumed correspondence between body and soul is nothing but a philosophical implementation of the mirror stage, by which the infant acquires a supposed identity and unity that originates in the Other of the signifier, the dit-manche. (Verhaeghe, 2002, p. 126)

Psychoanalysis reveals that the allegedly most natural fact – the human body – is to a considerable extent an outcome of primary socialisation and initially imaginary-narcissistic and then imaginary-symbolic identifications. Lacan has convincingly shown the paradox of the alienation-bound production of our psychosomatic identities, whose meanings emerge in a masquerade of imaginary and symbolic masks. The “mirror stage” concept explains how we become somebody by adopting externally imposed ideal images. Without this necessary self-alienation and the ousting of the “body-in-pieces,” our egos would not find in our bodiliness a foundation stable and integrated enough to ensure their correct development.

The analysis of the mirror stage thus leads Lacan to restate the function of the Ego. It is the instance that serves both to hold the subject together and provide that centred consistency necessary to subjective life. But it is also what *hides* the subject’s original splitting. Hence misrecognition “constitutes the ego, the illusion of autonomy to which it entrusts itself” so as to become an autonomous subject […]. But Lacan goes further. This founding misrecognition is in fact the condition of possibility of the subject’s being able to grow into and know the world. Knowledge (*connaissance*) is predicated on misrecognition (*méconnaissance*), on mis-knowing oneself (*me-connaissance* in French: knowledge of self). Thus as Lacan firmed up in his 1951 restatement of his initial 1949 lecture, misrecognition, the alienation of the subject into imaginary identifications pinned onto the body, is nothing short of the condition of possibility for human knowledge. (Epstein, 2016, pp. 20–21)

Therefore, the external image, in the semblance of which our body schema changes, is primarily outlined by the social environment, meaning that not only our minds but also “our” bodies are produced by others and the Other.

This reversal of perspective takes us beyond anthropology and extends the exploration field onto the social behaviour of animals. In conjunction with this, Lacan drew on experiments with female pigeons which found an evident interdependence between an individual’s biological development and the arrangement or its interindividual life. These experiments showed that without a visual reference to other members of its species, an identification-deprived female pigeon was unable to develop mature gonads. However, as soon as its species-image – its photographed or drawn “alter ego” – was placed within its sight, the situation changed diametrically and its body followed regular development.

Phenomenologically and enactivistically inspired neurology offers similar conclusions. Such research has shown that modifications of the subjectively recognised or unconsciously experienced body image not only trigger changes in the sensory, proprioceptive body schema but also transform its materiality by charting a new map of neurological relations between the brain and the entirety of the knowing and experiencing body. As Nicola Diamond noticed:

The brain-body map is “normally” influenced by sensory input derived from the interaction between body and environment. However, in cases where there is a loss of a limb, and therefore fresh sensory input is lacking, treatment using artificial limb and trickery with mirrors can activate the action–body memory and the mirror neurons, thereby creating a “corrective” corporeal image-sensory feedback mechanism which can reduce lower limb phantom pain and thus alter sensory perception of pain […]. This illustrates how the visual image derived from the environment can influence the construction of the brain–body map. (Diamond, 2013, p. 22)

To return to Lacan’s psychoanalysis, despite its moments of alienation, the narcissism of primary identification with the imaginary ideal of the Self, which takes place in the “mirror stage,” exerts an extraordinary influence on a developing individual. First and foremost, it prevents the emerging subject from instinctual regression, protects it against the lethal return of the body-in-pieces and shields it against excessive, perverse pleasure (*jouissance*), in which the Ego can be disintegrated by the overpowering Id.

Hardly coincidentally, it is after the identification with the specular illusion of complete self-mastery that muscle co-ordination dynamically develops in a child’s body. Still, in this subject-forming process, it is crucial that the development of libidinal “anatomy” and instinctual expression comply with the body ideals endorsed in a given culture. In case of misalignment, the subject is prone to serious psychosomatic disorders, as external acceptance and the sense of abiding by the rules of the symbolic order are utterly indispensable to it.

Narcissistic admiration alone is not enough; a child seeks acceptance form Others and turns his/her head away from the mirror toward adults in an attempt to make sure that his/her bodiliness, which is shaping up so tempestuously, looks and moves correctly. Briefly, s/he expects a symbolic anointment. In this way, “wound up” by the Big Other, we start to move in compliance with its will.

## The Aestheticisation of Movement as the Source of Free and Flexible Self-Development

An immovable environment rather quickly ceases to be an object of our conscious perception; however, as soon as a new wave of motion rises in it and rushes toward us, this re-invigorated environment opens up to our interiority as a strange and untamed exteriority which may become our world. For this to indeed come to pass, the subject must become psychophysically synchronised with the disparate rhythms and the surprising expression of that which is happening around, the way a brilliant artist does. The subject must perform properly matched movements to respond to the surrounding motions, whether those of an animal dashing by, a whizzing ball flung apparently from nowhere, or a dazzlingly flamboyant pas performed by his/her dancing partner; the subject’s e-motion will develop correctly if, and only if, s/he manages to harmonise with external movements. This ontological choreography is always based on anticipation, in which we take the risk and attempt to guess the intentions of the other party. Of course, in the case of moving objects, this is happens by virtue of naive personification or deification, where we attribute – as a rule unwittingly and against critical rationalism – intentional action to these things. In a sense, absolutely passive objects – that is, those devoid even of the illusion of movement that our imagination can ascribe on them, or, in other words, objects which do not “attract” our sight altogether, do not “radiate” through us and do not pierce us with their gaze – do not stimulate us to engage in a somaesthetic game. We do not co-construct a sensory space of symbolic encounter with them since, emptied of meaning, as they are, they fade into the background or, at best, function as useful handled tools which we manipulate “blindly,” as it were. However, as Martin Heidegger taught us, authentic experience, particularly in digitalised reality, is premised on the capacity to aesthetically experience unhandy reality, whose movements we are never able to fully foresee and which is a value in and of itself, one more real than our selfishly narcissistic phantasms.

Wherever humans are the agents of movement, the mechanisms of mirror identification obviously intensify. This phenomenon can be fruitfully interpreted through the lens of three quite divergent theoretical frameworks: the phenomenology of the body, neurology (mirror neurons), and psychoanalysis (mirror stage). In their different ways, all these disciplines show that by observing purposeful action we take over its externalised motion and embody it, thereby becoming to a considerable degree its mimetic continuation. The subject can avoid mechanical imitation by developing a profound awareness of these processes. Space for self-fashioning is fostered by reflection on the symbolic meanings of the moving mode we reproduce in which purely functional values are combined with mostly unconscious somaesthetic manifestation. If we select appropriate somaesthetic models and modify them in our unique ways, we can create our own distinctive style, at least to a certain extent.

Whatever style it should be, somaesthetic education through movement always teaches one how to experience aesthetic values, such as flexible unity, dynamic harmony, fluid beauty, and sublime (non)identity in a rational and embodied way. Such psychosomatically experienced values may become attributes of the self-fashioning subject.

This takes place, for example, when we see a bird flying at full speed rush past our head as a feathery ball that does not in the least resemble an encyclopaedic image and definition. We do not experience any cognitive disappointment in such a moment. Rather, captivated by the bird’s mind-boggling agility, we hasten to remove a net hung over the fence from its flight path and, relishing its litheness, we ascend into the sky with it. We are even more susceptible to somaesthetic fascination when we see moving people enveloped in an aura of soulful sensuality; at such moments, we crave to be infected with their magnificent personalities, meticulously imitating their facial expressions, gestures, and movements.

As the two interpretations above – phenomenological and Lacanian – show, the body-in-motion can be the source of both authentic and alienated subjectivity. Any attempts at conclusively eliminating this contradiction are doomed to failure in advance. A living body in the real world of other subjects builds its sense of agentive and receptive distinctiveness by exclusively relying on temporary boundaries. Where exactly these boundaries lie is determined by the dynamics of mutual interactions between changing cultural criteria, relatively stable intra-species norms, and individual psychological factors. If there is no petrified model of bodily subjectivity, there can be no fundamentalist approach to this issue.

The mind is known to avail itself of intuition whenever the unambiguous and ostensibly independent logic of the intellect proves insufficient. This mode of knowledge, which is unclear by default, can be somewhat specified, at least as much as to be effectively used to maximise subjective possibilities. To do so, we must shift our ponderings from intellectual abstractions into the realm of aesthetics and reflect on the cognitive potential of sensory perception and sensation, primarily focusing on the notion that there is a meaningful link between the sensorily embodied truth and the experience of harmonious completeness.

The aesthetic approach to the subjective meaning and significance of bodily movement and kinaesthesia is anti-fundamentalist in that, instead of inquiring about their role in producing an allegedly substantially permanent essence of a subject, it investigates their utility in individual development understood as a freely designed and dynamically harmonised project. What matters in this project is not who one is, but in what different ways one can effectively be. Given this, the alternative of authenticity vs. alienation becomes a secondary, and sometimes even an artificial, issue, the problem of which is solved by the pragmatic approach to bodily movement and kinaesthesia, which seeks to grasp their real utility – their temporary and self-correcting effectiveness. In this quasi-artistic framework, a human being is conceived as an open work of art. Nothing promotes self-experience as a kaleidoscope of wonderful and compelling possibilities more efficiently than experiencing this directly and primarily through bodily self-motion, when the body does not act in order to achieve an externally defined aim, but enjoys self-motion as an embodied confirmation of its own impressively free agency.

Underscoring their epistemological openness and unconventional pragmatism, which have often yielded remarkable results as evinced, for example, by Merleau-Ponty’s and Lacan’s superb writings, both parties to the discussion on the subject-forming function of bodiliness – phenomenology and psychoanalysis – have often settled central issues by drawing on aesthetic explorations and exemplifications borrowed from artistic theories and practice.

John Dewey was one of the first and at the same time most important theorists to grant aesthetic experience a fundamental role in experiencing as such. Dewey, an American psychologist and philosopher whose work has inspired considerable interest among phenomenologists and psychoanalysts, rejected both the sterile, abstract ways of thinking and the mechanist reductionism of utterly materialistic empiricism. Instead, he proposed developing knowledge capable of mirroring the incessantly changing, living reality of experience, a knowledge which did not sever defining thought from feeling body.

A fully experiencing human being resembles an artist who filters the surfeit of sensations to capture those which open up his/her experience onto the world in the aesthetic feeling of reconciliation and beautiful harmony. In his/her pursuit of the classical order, this aesthete seeks to manage, if not to eliminate, negative impressions, whether they come from him/herself or from the world outside, for if experience is dissonantly split, both the world and his/her own existence elude him/her.

According to Dewey, the role of aesthetic emotion in experience is that “[i]t selects what is congruous and dyes what is selected with its colour, thereby giving qualitative unity to materials externally disparate and dissimilar. It thus provides unity in and through the varied parts of an experience” (Dewey, 2005, p. 23).

This need for aestheticisation concerns any experience, including when its generic destiny does not lie in realising a positive aesthetic quality, since it can involve purely cognitive values, as in theoretical or scientific experience, as well as practical values associated with utility in a broad sense of the term. Nonetheless, whatever the type of experience, it demands establishing a harmonious relationship with the environment. As Dewey insisted, only when aesthetically integrated in this way does experience become “an experience” – genuine and complete.

At the moment, “an experience” conceived in this way is mostly championed by enactivist, who actively advocate the embodied mind and have worked to liberate contemporary cognitivism from the “brain-in-the-vat” illusion since the 1990s. In their interpretations of research findings on the neural structures of cognition, enactivists explain that experience deeply depends on sensorimotor patterns, which are shaped through interactions with the natural environment. They insist that aesthetically positive emotions, which underlie sensorimotor habits, foster the fullest and thus most satisfying interaction with the world and with other subjects. Such views are espoused, for example, by Ioannis Xenakis and Argyris Arnellos:

There are several recent neurological evidences that support this hypothesis. Relevant studies have showed that there are several operations that are simultaneously taking place in various interconnected areas of the human brain during an aesthetic experience, in particular, or/and during other anticipative/evaluative interactions in general. These studies suggest that humans anticipate the impact of future behavioral choices on the basis of reward values, using processes that involve the amygdala, which is mostly known for emotional processing during an aesthetic experience […], as well as areas in the prefrontal cortex (PFC) […]. Moreover, both the amygdala and the orbito-frontal cortex (OFC), which is also activated in most of the studies related to aesthetic experiences […], are extremely well positioned to tune perceptual processing in sensory cortex based on stimulus evaluation […]. Dysfunction of OFC is associated with disturbances in motivation and an inability to anticipate interactive consequences, leading to maladaptive behavior. (Xenakis and Arnellos, 2015, pp. 253–254)

Besides brain activity, our perception and sensory sensations need an experiencing and experienced body whose kinaesthetic motivation underlies perspectival knowledge; through moving in the sense environment, the body reveals various profiles and manifestations of object and subjects it encounters. Which of the perspectives made available to us by objects of experience most completely render the contextual essence of things is communicated to us by the aesthetic feeling of harmonious synchronisation between the subject and the object of knowledge.

All forms of knowledge, including knowledge conveyed in language, derive their content and forms from carnal sources, especially one as vigorous and exploring as the body-in-motion. For this reason, a careful and deliberate cultivation of the body and perfecting its aesthetic awareness are invariably paramount human tasks. These tasks are all the more critical today, as the increasingly technologised and industrialised world we inhabit exposes us to intensifying anaesthetisation. As a result of the ubiquity of digital media, we are losing our natural sensitivity, and consequently when “the body as a locus of sensory-aesthetic appreciation (aesthesis) and creative self-fashioning” (Shusterman, 2012, p. 27) lets us down, the pathological inability to understand oneself and other people spreads.

Therefore, we must work to re-establish the natural bond with our bodies if we want to avoid losing contact with humanly conceivable reality. The Greek unity of beauty, truth, and good – an ideal which until recently was evidently dominant in our culture – calls for a new embodiment. We must entrust not only our minds but also our bodies to aesthetic education, aesthetic rehabilitation, and aesthetic therapy.

Today, the virtualisation of the body causes individuals to struggle as they are looking for a convincing self-reflection in the media deluge of changing and flickering images. The surfeit and the provisionality of nagging patterns breed fears characteristic of “borderline” personality in confused individuals. Disorders, such as the sense of bodily alienation and uncertainty in recognising and experiencing one’s own psychosomatic boundaries, are no longer typical solely of puberty (Lemma, 2017). Across age groups, people are generally jeopardised by the medially simplified alter-ego images. Without finding a reflection in real-life conditions in real embodied Others, without being mirrored in the symbolic “flesh-and-blood” counterparts, the subject who is uncertain of his/her identity falls back on psychotic defensive self-identifications, donning tight costumes of cybernetic avatars (Lemma, 2015).

Proper self-reflection in imaginary others and then in symbolic Others is a necessary condition of subjective development and of an elementary sense of self-identity, a condition which we begin to meet the moment we are born. Intense communication between the child and its parents takes place even before the acquisition of proper language competencies. As Dana Shai and Peter Fonagy put it, this

Parental embodied mentalizing (PEM) refers to the parental capacity to (1) implicitly, and not necessarily consciously, conceive, comprehend, and extrapolate the infant’s mental states (such as wishes, desires, or preferences) from the infant’s whole body kinaesthetic expressions, namely changes in body movement and posture and (2) adjust their own kinaesthetic patterns accordingly. Importantly, and reflecting a relational perspective, the parental kinaesthetic behaviours are not considered in isolation, but always in reference to those of the infant. (Shai and Fonagy, 2013, p. 60)

## Conclusion

To keep things feasible, we focus on ourselves to one fundamental demand that indicates the goal of the possible methodology. Whichever specific techniques are used in research on the influence of movement on the subject’s consciousness and whichever method is adopted, it should seek to capture the symbolic and mental meanings of motion-induced affects. Regrettably, sundry therapies of the Awareness-Through-Movement type ignore the symbolic dimension of affect and do not explore it in the context of individuals’ identity histories.

Affects are never thoughtless. Therefore, body awareness therapy should not cultivate “experience for the sake of experience.” This may breed narcissistic and psychotic disorders, instead of self-knowledge and self-development. The body must not play the role of a stammering “ventriloquist.” Thus, if body and mind form a dynamic whole, our research must be wary of both one-sidedly abstract intellectualism and the fundamentalistically unreflecting carnalism (Soler, 2016).

In psychotherapy, physiotherapy and, likewise, art therapy, we deal with somatic disorders which clearly arise in conjunction with deficits in mentalising capacities. Patients cannot express their sensations, feelings, and affects. It turns out that their therapies are considerably aided by interventions, methods, and means that bolster their capacity to aesthetically experience their own psychosomatic setup as a harmoniously integrated whole.

Reflection and the transformation of the subject’s affective dimension are best fostered by therapies that employ aesthetic methods and means, combining artistic activities with somaesthetics theory and practice underpinned by physical exercises (Bloom, 2006; Fedorova, 2020). There is nothing as convincing as the intimate, palpably embodied truth when its meaning is revealed simultaneously in kinaesthetic, tactile, and emotional moments in the form of a harmonious experience of completeness. Experienced in this way, the subject more readily integrates into a meaningful whole. Moreover, the artistically attuned subject can design, invent, and enact him/herself by manipulating his/her own capacities in the semblance of artistically sampled elements of a constantly developing artwork. Such an aesthetic of the body which is being experienced kinaesthetically stands in stark contrast to the currently spreading “Botox aesthetics,” in which the body image is entirely separated from the body schema. This difference is vividly conveyed by the paralysed and almost expressionless faces of a more and more numerous throng of actors and actresses who, in order to continue in their jobs, have exchanged the human face, an instrument endowed with a limitless articulation palette, for a facially congealed mask, a caricature of thoughtless “beauty” detached from the rest of the gesticulating and moving body. To continue this histrionic metaphor, the body image should always surface to the visible level as an emanation of deep motions, as a manifestation of a buoyantly and harmoniously lived life. On the stage of life, the lead role must be played by an aesthetically moved and self-moving dynamic unity of body-mind (or mind-body).

## Data Availability Statement

The original contributions presented in the study are included in the article/supplementary material, further inquiries can be directed to the corresponding author.

## Author Contributions

RD and KP contributed to conception and design of the study. Both authors reviewed the results and approved the final version of the manuscript.

## Conflict of Interest

The authors declare that the research was conducted in the absence of any commercial or financial relationships that could be construed as a potential conflict of interest.

## Publisher’s Note

All claims expressed in this article are solely those of the authors and do not necessarily represent those of their affiliated organizations, or those of the publisher, the editors and the reviewers. Any product that may be evaluated in this article, or claim that may be made by its manufacturer, is not guaranteed or endorsed by the publisher.

## Footnotes

1 Though this may not be immediately recognisable to modern speakers, *equivalent* is derived from *valere* and *valentia* (Latin: to be strong, strength).

2 Though less directly noticeable, this linguistic kinship is also intrinsic to the English word “concept,” which stems from Latin *concipere* – to take in and hold, to become pregnant.

3 The Big Other is capitalised in order to emphasise that it is distant and inaccessible and to differentiate it from “others” that are basically “the same” as us. For example, for a believer God is the Big Other and other people are others.

4 Of course, it is not only about the separation from the biological mother; this role can be played by another caregiver as well.

## References

[B1] Alpha R. Hurst J. (2018). Collapsing the surfaces of skin and photograph in cosmetic minimally-invasive procedures. *Body Soc.* 24 175–192. 10.1177/1357034x18766289

[B2] Bloom K. (2006). *The Embodied Self**: Movement and Psychoanalysis.* London: Karnac.

[B3] Bower T. G. R. (1971). The separation of place, movement and object in the world of the infant. *Sci. Am.* 225 30–38. 10.1038/scientificamerican1071-30 5106748

[B4] De Preester H. Knockaert V. (2005). *Body Image and Body Schema: Interdisciplinary Perspectives on the Body.* Amsterdam: Ghent University. 10.1075/aicr.62

[B5] Dewey J. (2005). *Art as Experience.* New York: Penguin Publishing Group.

[B6] Diamond N. (2013). *Between Skins: The Body in Psychoanalysis – Contemporary Developments.* Chichester: Wiley-Blackwell. 10.1002/9781118321102

[B7] Epstein C. (2016). Surveillance, privacy and the making of the modern subject: habeas what kind of corpus? *Body Soc.* 22 28–57. 10.1177/1357034x15625339

[B8] Evans D. (2006). *An Introductory Dictionary of Lacanian Psychoanalysis.* London: Routledge. 10.4324/9780203135570

[B9] Fedorova K. (2020). *Tactics of Interfacing: Encoding Affect in Art and Technology.* Cambridge, MA: The MIT Press. 10.7551/mitpress/12544.001.0001

[B10] Foucault M. (1977). “Nietzsche, genealogy, history,” in *Language, Counter-Memory, Practice: Selected Essays and Interviews*, ed. Bouchard D. F. (Ithaca, NY: University Press), 153.

[B11] Husserl E. (2000). “Ideas pertaining to a pure phenomenology and to a phenomenological philosophy,” in *Edmund Husserl Collected Works*, Vol. III, ed. Stein E. (Dordrecht: Kluwer Academic Publishers), 273.

[B12] Lacan J. (1988). *The Seminar. Book I. Freud’s Papers on Technique, 1953–54.* Cambridge: Cambridge University Press.

[B13] Lacan J. (2006). “The mirror stage as formative of the function of the I,” in *Ecrits: The First Complete Edition in English*, eds Lacan J. Fink B. (New York, NY: W. W. Norton & Company), 75–81.

[B14] Lakoff G. Johnson M. (1999). *Philosophy in the Flesh: The Embodied Mind and Its Challenge to Western Thought.* New York, NY: Basic Books.

[B15] Lemma A. (2015). *Minding the Body. The Body in Psychoanalysis and Beyond.* London: Routledge. 10.4324/9781315758824

[B16] Lemma A. (2017). *The Digital Age on the Couch Digital. Psychoanalytic Practice and New Media.* London: Routledge. 10.4324/9781315212524

[B17] Meltzoff A. N. Moore M. K. (1983). Newborn infants imitate adult facial. *Child Dev.* 54 702–709. 10.2307/1130058 6851717

[B18] Merleau-Ponty M. (1964). “The child’s relations with others,” in *The Primacy of Perception*, eds Merleau-Ponty M. Edie J. M. (Evanston: Northwestern University Press), 96–155.

[B19] Noë A. (2004). *Action in Perception.* Cambridge, MA: MIT Press.

[B20] Reich W. (1945-1972). *Character Analysis.* New York, NY: Touchstone.

[B21] Sacks O. (1998). *The Man Who Mistook His Wife For a Hat and Other Clinical Tales.* New York, NY: Simon & Schuster.

[B22] Scarinzi A. (2015). “Enactive literariness and aesthetic experience: from mental schemata to anti-representationalism,” in *Aesthetics and the Embodied Mind: Beyond Art Theory and the Cartesian Mind-Body Dichotomy*, ed. Scarinzi A. (Dordecht: Springer), 266. 10.1007/978-94-017-9379-7_16

[B23] Shai D. Fonagy P. (2013). “Beyond words: parental embodied mentalizing and the parent infant dance,” in *Nature and Formation of Social Connections: From Brain to Group*, eds Mikulincer M. Shaver P. R. (Washington, DC: American Psychological Association Press), 60. 10.1037/14250-011

[B24] Sheets-Johnstone M. (2011). *The Primacy of Movement.* Amsterdam: John Benjamins Publishing Company. 10.1075/aicr.82

[B25] Shusterman R. (2012). *Thinking Through the Body: Essays on Somaesthetics.* Cambridge: Cambridge University Press. 10.1017/CBO9781139094030

[B26] Soler C. (2016). *Lacanian Affects. The Function of Affect in Lacan’s Work* (B. Fink, Trans.). London: Routledge. 10.4324/9781315731797

[B27] Stern D. N. (1985). *The Interpersonal World of the Infant: A View from Psychoanalysis and Developmental Psychology.* New York, NY: Basic Books.

[B28] Verhaeghe P. (2002). “Lacan’s answer to the classical mind/body deadlock,” in *Reading Seminar XX: Lacan’s Major Work on Love, Knowledge, and Feminine Sexuality*, eds Barnard S. Fink B. (Albany, NY: State University of New York Press), 126.

[B29] Weigel S. Scharbert G. (2016). *A Neuro-Psychoanalytical Dialogue for Bridging Freud and the Neurosciences.* Heidelberg: Springer. 10.1007/978-3-319-17605-5

[B30] Xenakis I. Arnellos A. (2015). “Aesthetics as an emotional activity that facilitates sense-making: towards an enactive approach to aesthetic experience,” in *Aesthetics and the Embodied Mind: Beyond Art Theory and the Cartesian Mind-Body Dichotomy*, ed. Scarinzi A. (Dordecht: Springer), 253–254. 10.1007/978-94-017-9379-7_15

